# The effects of a web-based computer-tailored diet and physical activity intervention based on self-determination theory and motivational interviewing: A randomized controlled trial

**DOI:** 10.1016/j.invent.2022.100537

**Published:** 2022-04-14

**Authors:** Juul M.J. Coumans, Catherine A.W. Bolman, Anke Oenema, Lilian Lechner

**Affiliations:** a Department of Health Psychology, Faculty of Psychology, Open University of the Netherlands, Heerlen, the Netherlands; b Department of Health Promotion, Care and Public Health Research Institute (CAPHRI), Faculty of Health, Medicine and Life Sciences, Maastricht University, Maastricht, the Netherlands

**Keywords:** eHealth, Self-determination theory, Motivational interviewing, Diet, Physical activity, Randomized controlled trial, Effectiveness

## Abstract

**Background:**

According to self-determination theory (SDT), autonomous forms of motivation are more likely to result in sustained behavioral changes than controlled forms. Principles of motivational interviewing (MI) can be applied to facilitate more autonomous forms of motivation. This study investigated whether a combined diet and physical activity (PA) web-based computer-tailored intervention based on SDT and MI, called MyLifestyleCoach, was effective in promoting dietary and PA behaviors.

**Methods:**

A two-arm randomized controlled trial with 1142 Dutch adults was conducted. The intervention and control group completed questionnaires at baseline, 6, and 12 months from baseline. Only participants in the intervention condition had access to MyLifestyleCoach. The waiting list control condition had access to the intervention after completing the 12-month follow-up questionnaire. A modified food frequency questionnaire was used to measure dietary behaviors (fruit, vegetables, fish, and unhealthy snacks). The Dutch Short Questionnaire to ASsess Health was used to measure the weekly minutes of moderate-to-vigorous PA (MVPA). Usage data, which is operationalized as completed sessions in this study, was objectively assessed by log data. We conducted two-step linear mixed effect models. In the first step, a model consisting of condition, time, potentially confounding variables and a random intercept for participants was tested. In the second step, an interaction term was added to investigate the intervention's (time × condition) and usage (time × opening session and time × completed sessions) effects over time for the dietary and PA outcomes.

**Results:**

The findings showed no differences between the groups for all four dietary behaviors and the weekly minutes of MVPA at any of the time points. In-depth analyses showed that participants who followed the opening session of the intervention, in which they received personalized feedback on their behaviors, had a stronger increase in fruit consumption at 6 months and 12 months than participants who did not follow the interventions' opening session. Lastly, participants who followed more sessions in the diet module had a stronger increase in fruit and vegetable consumption at 6 months, and a stronger decrease in the consumption frequency of unhealthy snacks at 12 months post-baseline.

**Conclusion:**

Overall, the intervention was not effective in changing dietary and PA behavior. However, moderation analyses suggest that the intervention is effective in changing dietary behavior for those participants who used the intervention more intensively. Further research should focus on improving intervention use.

## Highlights

• The study investigates the effects of a web-based computer-tailored diet and PA intervention based on SDT and motivational interviewing.
• No overall effects of the intervention compared to a wait list control condition were found on dietary and physical activity outcomes.
• When intervention usage was considered, the intervention was promising in changing fruit and vegetable intake, and snacking behavior.

## 1 Background

Lifestyle behaviors can significantly impact health (Farhud, 2015). Two modifiable key healthy lifestyle behaviors are a healthy diet and regular physical activity (PA). These behaviors contribute to better mental well-being and lower the risk of non-communicable diseases, such as cardiovascular disease, diabetes, and certain types of cancers (GBD 2013 Mortality and Causes of Death Collaborators, 2015; Walsh, 2011). Many individuals worldwide have one or several unhealthy lifestyle behaviors. These lifestyle behaviors often tend to cluster, making people who engage in unhealthy dietary and PA behaviors more vulnerable to developing non-communicable diseases than individuals who engage in healthy behaviors. Therefore, it is essential to promote these multiple lifestyle behaviors in an integrated manner (Edington, 2001; Fine et al., 2004). Interventions based on individual health coaching have shown promise in changing several lifestyle behaviors (Olsen and Nesbitt, 2010). Unfortunately, one-to-one, and in particular, face-to-face counseling is intensive, costly, and only suitable to reach a limited number of people.

Computer tailoring (CT) is a technique that can combine individual counseling with a large reach (Kreuter and Skinner, 2000). CT provides individual feedback that matches personal characteristics and needs, through an automated system, making it a suitable technique for combining individual counseling with a largescale reach at relatively low costs (Neville et al., 2009; Lustria et al., 2009). Systematic reviews and meta-analyses demonstrate that CT interventions (either web or paper-based) can be effective in modifying dietary and PA behavior (Kroeze et al., 2006; Davies et al., 2012; Teasdale et al., 2018; Neville et al., 2009). However, these interventions generally have small and only short-term effects.

So far, most of these CT interventions have been based on traditional health behavior theories, such as the transtheoretical model, the social cognitive theory, and the health belief model (Lustria et al., 2013). They use a rational, cognitive, and directive approach by convincing people of the importance of eating healthily, becoming more active, and complying with recommendations. These approaches primarily inform about the importance of complying to the guidelines, whereby the types of motivation and people's own interests are less considered. According to the self-determination theory (SDT), autonomous forms of motivation, particularly intrinsic motivation, are more likely than extrinsic forms to result in sustained behavior change (Teixeira et al., 2011, Teixeira et al., 2012). Several studies have shown that intrinsic motivation, which is a form of autonomous motivation, has been related to healthier eating patterns and the long-term adoption of PA (Hagger and Chatzisarantis, 2008; Verstuyf et al., 2012). Researchers have proposed that a context fostering the basic psychological needs of autonomy, relatedness, and competence leads to the development or the maintenance of autonomous forms of motivation that subsequently results in behavior change (Ryan and Deci, 2000; Deci and Ryan, 2008; Sheeran et al., 2020; Markland et al., 2005). The widely adopted motivational interviewing (MI) counseling style can provide the strategies needed to create such a stimulating environment (Miller and Rollnick, 2013; Markland et al., 2005). Thus, CT interventions based on SDT/MI could be promising and more effective than CT interventions based on the more traditional health behavior models in changing behavior and maintaining change.

Previous (clinical) trials based on the SDT/MI framework have proven their efficacy in changing motivation and health behaviors (Martins and McNeil, 2009; Burke et al., 2003). However, most interventions targeted only one health behavior, often PA, or were based on one-to-one counseling, such as face-to-face or by telephone, thereby having a small reach (Norman et al., 2007; Shingleton and Palfai, 2016; Gillison et al., 2019). Web-based CT interventions based on SDT/MI are relatively scarce, but the SDT/MI approach may be a promising approach for CT web-based health promotion as well. One web-based CT intervention based on SDT/MI that was developed by our research group, was effective in changing PA behavior (Friederichs et al., 2015, Friederichs et al., 2016). In that study, this new theoretical approach was compared with an intervention based on traditional health behavior theories such as the Social Cognitive Theory, and a no-intervention control group. Yet, it is unknown whether this approach can also be effectively implemented for healthy eating in a combined PA and dietary intervention.

The main aim of this study is to evaluate the effects of a web-based CT diet and PA promotion intervention based on SDT/MI in the short term (6 months after baseline) and long term (12 months after baseline) on dietary and PA behavior. We focused on the effectiveness of this intervention as a whole, not on the added value of SDT/MI as done previously by Friederichs et al., 2015, Friederichs et al., 2016. Further, we focused on combing diet and PA behavior instead of only focusing on PA behavior. We hypothesized that participants in the intervention condition compared to the control condition would increase their consumption of fruit, vegetables, and fish, and decrease their frequency of eating unhealthy snacks over time. Furthermore, we hypothesized that participants in the intervention condition would increase their weekly minutes of moderate-to-vigorous PA (MVPA) at 6 and 12 months compared to the participants in the control condition (Vansteenkiste and Sheldon, 2006; Rubak et al., 2005; Friederichs et al., 2015, Friederichs et al., 2016). We also expected that effects would be more pronounced for those who used the intervention more intensively.

## 2 Methods

### 2.1 Participants

Eligible participants were Dutch adults, aged between 18 and 70, with an adequate understanding of the Dutch language, and who had access to a computer/tablet with access to the Internet. Participants who indicated to have already participated in previous comparable studies of our research group were excluded. Participants were recruited among members of an online research panel between October 2018 and May 2019. This panel consists of people with various socio-demographic characteristics varying in age, sex, educational level, working status etc. who are interested in participation in online studies and who are willing to participate without receiving a fee. Although the panel consisted of voluntary members who do not receive fees from the research panel, for the current study we did provide lottery prices among those who completed all questionnaires or intervention parts. This randomized controlled trial (RCT) has been ethically approved by the Committee for Ethics and Consent in Research of the Open University of the Netherlands (reference number: U2018/07266/SVW) and was registered in the Dutch Trial Register (NL7333).

### 2.2 Study design

An RCT was conducted in which participants were allocated to the MyLifestyleCoach intervention condition or to the waiting list control group. Measurements were taken at baseline, and 6- and 12-months post baseline.

### 2.3 Procedure

#### 2.3.1 Recruitment

A research panel sent several e-mails to recruit participants for this study. In this e-mail, some basic information was provided about this study, and participants then could choose to click on a link leading them to the study website with additional information. If participants wanted to start, they could click on the “I want to participate button”.

#### 2.3.2 Preliminary Assessment and Baseline Questionnaire

First, potential participants had to fill in some questions to assess the previously described inclusion/exclusion criteria of this study and had to sign an informed consent. After that, participants were randomly assigned in a computer determined sequence to the intervention condition or the waiting list control condition and filled in the baseline questionnaire that took about 30–45 min to complete. A ratio of 2:1 was chosen to retain enough participants in the intervention to obtain sufficient power to conduct the analyses.

#### 2.3.3 Intervention

**Fig. 1:**
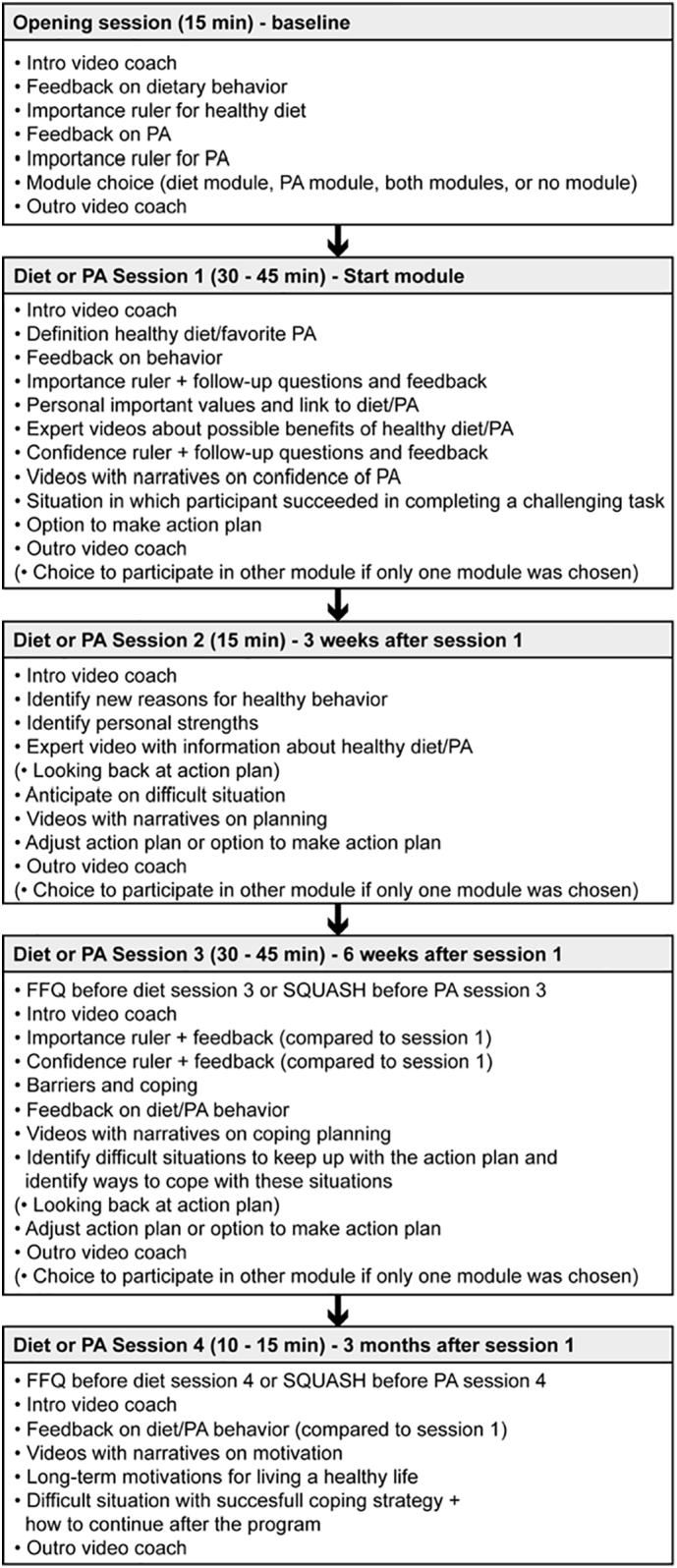
Overview of the content of the sessions in the intervention.

MyLifestyleCoach is a web-based CT intervention that consists of a general opening session, followed by a diet module to promote dietary behavior and a previously tested PA module to improve PA levels of Dutch adults (Friederichs et al., 2015, Friederichs et al., 2016). This intervention is based on principles of SDT and uses counseling techniques derived from MI. The intervention has been developed using the Intervention Mapping protocol (Bartholomew Eldrigde et al., 2016). Detailed descriptions of the development of this intervention were published previously (Coumans et al., 2020a; Friederichs et al., 2014). Here we provide a short overview about the intervention's content (see also Fig. 1).

Participants in the intervention continued to the opening session once they completed the baseline questionnaire. Here, they were introduced to the program and to the video coaches that would guide them through the program. They also received individual feedback on their dietary and PA behaviors using a traffic light system based on the results of the baseline questionnaire. The color of the traffic light indicated whether they complied to a guideline (green light), there would be some room for improvement (orange light) or whether there would be a lot of room for improvement (red light). Based on this feedback, participants were advised which modules (diet and/or PA) to use (for more details see (Coumans et al., 2020b). In compliance with the SDT principles, the traffic lights were purely meant to provide participants with an insight into what they could change and were not necessarily intended to induce compliance with PA guidelines and dietary recommendations. Participants were free to choose their own goals within the intervention. Furthermore, they rated the personal importance of a healthy diet and sufficient PA on an importance ruler ranging from 1 to 10. At the end, they could choose which of the modules they would like to take part in: both modules, the diet module only, the PA module only, or no module; and when to start with the module(s), directly or at a later moment.

Both the diet and PA modules consisted of four sessions in which different topics were addressed. The first session took place immediately after the opening session or at a chosen point within 2 weeks after the opening session depending on whether participants choose only one or both modules. See Fig. 1 for the timeline and duration of the sessions. Participants did not have to complete a session before getting access to the following session. The topics of the modules were similar in structure for the diet and PA intervention but both were applied to diet or PA, depending on the participant's module choice. Before session 3 and 4 participants were also asked to fill in a questionnaire regarding diet or PA behavior. The results on these questionnaires served as input for feedback in the sessions.

#### 2.3.4 Waiting list control condition

Participants allocated to the waiting list control condition had no access to the intervention. After the 12-month study period, i.e., when they completed the 12-months questionnaire, they were given access to the intervention.

#### 2.3.5 Follow-up questionnaires

Six and 12 months after baseline, the participants were sent an invitation e-mail to complete the follow-up questionnaires that took a maximum of 40 min to complete. e-Mail reminders were sent every week for 4 weeks in total. All measurements were taken by web-based questionnaires via the study website.

### 2.4 Measurements

The baseline questionnaire assessed demographic characteristics, dietary and PA behavior, and psychosocial constructs. All measurements were self-reported.

#### 2.4.1 Demographics

Demographic characteristics included age, gender, education (recoded into three categories: low, middle, and high), work status (employed and unemployed), physical impairment (yes/no), marital status (single or partner), weight and height that were used to calculate BMI (kg/m^2^), and health status using a 100-point thermometer-style visual analog scale. These factors served as control variables in our analysis.

#### 2.4.2 Dietary behavior

For this RCT, dietary behaviors focused on the consumption of fruit, vegetables, fish and the frequency of unhealthy snacks. These outcomes were assessed using an adapted Flemish validated Food Frequency Questionnaire (FFQ) (Huybrechts et al., 2011; Ahrens et al., 2016). In this questionnaire, participants had to fill in on how many days in a typical week in the last month (ranging from 0 to 7 days) they consumed fruit, vegetables, and fish. This FFQ was extended with questions about the size of fruit and vegetables portions based on Willems et al. (2015). The intake of pieces of fruit per day was calculated by multiplying the frequency by the number of pieces with the reported number of consumption days, divided by 7 (days a week). Vegetable consumption per day was calculated by multiplying the number of days on which participants reported to eat vegetables × number of portion sizes per day × 50, as one portion equals 50 g, divided by 7 (days a week). The consumption frequency of snacks was determined in the following way. Participants reported how many times in a typical week in the last month they consumed the following eight types of snacks: unsalted nuts, dried fruits, chocolate, candy, cookies, chips, ice cream, and savory pastries on 7-point Likert scale: 1 = never/less than once a week (0), 2 = 1 to 3 times a week (2), 3 = 4 to 6 times a week (5), 4 = 1 time per day (7), 5 = 2 times per day (14), 6 = 3 times per day (21), or 7 = 4 or more times per day (30). Examples were provided for each snack type. Some of these example Flemish snacks were changed by a Dutch alternative, as some of these snacks were not available, have a different name, or are not common in the Netherlands. The consumption frequency of unhealthy snacks per day was determined by summing the recoded frequencies, in parentheses, for the last 6 snacks (chocolate to savory pastries) divided by the numbers of days in a week. The dietary outcomes were thus daily fruit intake, daily vegetable intake, weekly fish consumption, and the daily consumption frequency of unhealthy snacks.

#### 2.4.3 PA behavior

PA behavior for a typical week in the past month was assessed using the validated Dutch Short Questionnaire to ASsess Health (SQUASH) (Wendel-Vos et al., 2003). PA behavior was operationalized as the total number of minutes of MVPA per week by multiplying the frequency (days per week), and duration (hours and minutes per day) of leisure and transport walking, leisure and transport cycling, work, household activities, gardening, odd jobs, and sports performed with moderate or vigorous intensity. According to the guidelines of the SQUASH, individuals who reported spending more than 6720 min on PA per week on any of the time points were excluded, as they were considered unreliable (31 observations).

### 2.5 Statistical analysis

Descriptive statistics (means, standard deviations (SD)) and frequencies (and percentages) were used to depict the characteristics of the participants. First, predictors of dropout were examined separately for 6 and 12 months using logistic regression analyses. Dropout was defined as did not answering any of the questions of the follow-up questionnaire. These predictors included demographic characteristics, condition, and dietary and PA outcomes and served as control variables in the subsequent analyses.

Subsequently, three sets of linear mixed-effects models using the maximum likelihood procedure were conducted. Each set started with a linear mixed effect model (basic model) consisting of the potentially confounding variables age, gender, education, work status, physical impairment, marital status, BMI, and health status. In the first set of linear mixed effect models, the interaction term (time × condition) was added next to condition and the measurements of the separate behaviors at respectively 6 and 12 months to investigate the intervention's effects over time for the dietary (fruit, vegetables, fish, and unhealthy snacks) and PA (weekly minutes of MVPA) outcomes as is conventional in a traditional RCT.

To explore the impact of intervention usage on the intervention effects in a second set of linear effect models, following the opening session and the time × opening session interaction were added as additional variables to the previous model. When participants reached the end of a certain session, a variable for that session was set at complete. The opening session is a specific and separate part of the intervention and differs from the other sessions. In this opening session participants receive feedback on their behaviors and can then make a choice for the diet and/or PA module or no module. As this session is crucial for the further trajectory within the intervention, the opening session was considered as a separate variable.

In the third set of linear effect models, session usage of the diet and PA module and time × number of sessions (0–4 sessions) interaction were added as variables to the previous basic model. The value of 0 was set for people in the wait list control condition or people who did not complete a module's session in the intervention condition. A random intercept for participants was included in all models. Continuous variables were centered before the analyses.

Visual inspection using histograms showed that the outcomes were skewed, and therefore these outcomes were transformed using the square root. The significance levels for interaction terms were set to *p* < .10 since they have less power (Twisk, 2006). All analyses were corrected for multiple testing using the Bonferroni adjustment. All statistical analyses were performed with the statistical software R (version 3.6.0) (R Core Team, 2013).

## 3 Results

### 3.1 Participant characteristics

**Fig. 2:**
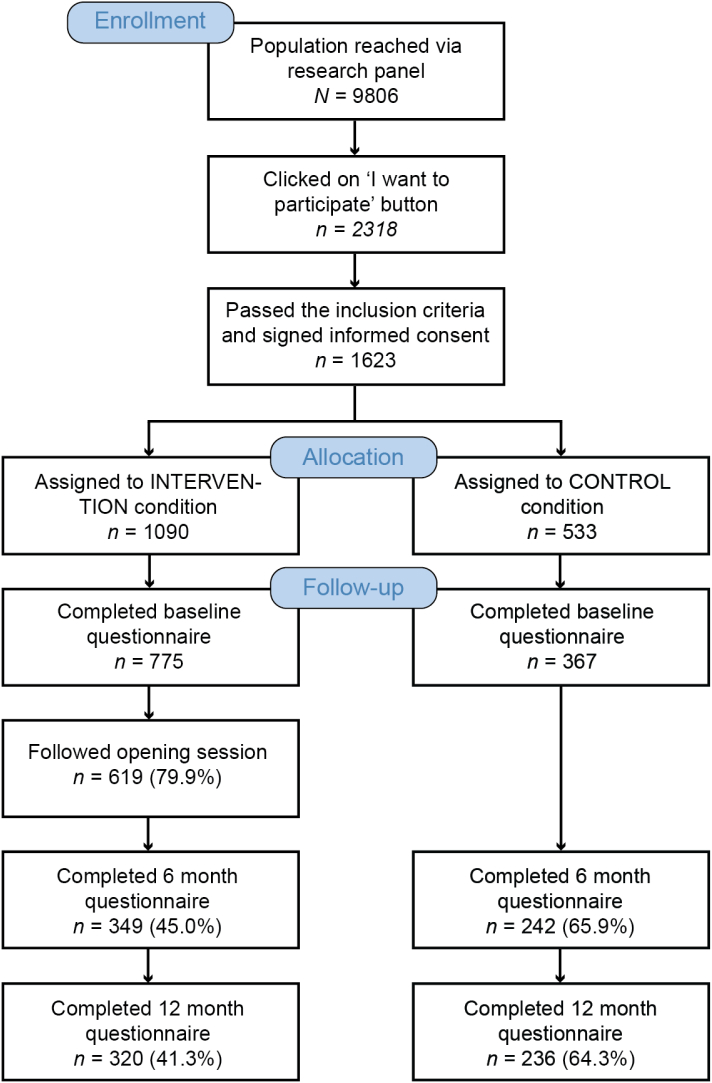
Flow of the participants in the RCT.

For this study, 9806 individuals were approached via an online research panel to participate in this study and 2318 (23.6%) clicked on the “I want to participate” button. In total, 1623 individuals were eligible, as they passed the inclusion and exclusion criteria (see methods section) and filled in the informed consent. Out of the 1623 eligible participants, 1142 (70.4%) participants completed the baseline questionnaire in this study, of which 775 in the intervention condition and 367 in the waiting list control condition (Fig. 2). In the intervention condition, 619 participants continued to the opening session where the received feedback on their dietary and PA behavior and made a choice on which module(s) to follow.

The mean age of the sample was 52.1 years old (Table 1). Almost 60% of the participants were women and about 70% of this sample had a high education. About two-thirds of the sample had a partner and were employed. The mean BMI of this sample was slightly overweight.

**Table 1:** Baseline characteristics of the study population.

Variables	Level	Intervention	Control	Total
*N*		775	367	1142
Age (years; mean (SD))		51.9 (13.1)	52.6 (12.9)	52.1 (13.0)
Gender (%)	Female	475 (61.3)	217 (59.1)	692 (60.6)
	Male	300 (38.7)	150 (40.9)	450 (39.4)
Education (%)	Low	29 (3.7)	20 (5.4)	49 (4.3)
	Medium	201 (25.9)	90 (24.5)	291 (25.5)
	High	545 (70.3)	257 (70.0)	802 (70.2)
Marital status (%)	Partner	529 (68.3)	248 (67.6)	777 (68.0)
	Single	246 (31.7)	119 (32.4)	365 (32.0)
Work (%)	Employed	496 (64.0)	250 (68.1)	746 (65.3)
	Unemployed	279 (36.0)	117 (31.9)	396 (34.7)
Physical impairment (%)	No	740 (95.5)	354 (96.5)	1094 (95.8)
	Yes	35 (4.5)	13 (3.5)	48 (4.2)
BMI group (%)	Underweight	15 (1.9)	4 (1.1)	19 (1.7)
	Normal	328 (42.3)	164 (44.7)	492 (43.1)
	Overweight	279 (36.0)	131 (35.7)	410 (35.9)
	Obese	153 (19.7)	68 (18.5)	221 (19.4)
BMI (kg/m^2^) mean (SD))		26.5 (5.2)	26.3 (5.1)	26.4 (5.2)
Health status (mean (SD))		69.9 (15.6)	70.1 (14.1)	70.0 (15.2)

*Note*. No significant baseline differences were found between the intervention and control group.

### 3.2 Dropout attrition

The logistic regression analyses assessing dropout for the 6- and 12-months follow-up questionnaires showed that participants in the intervention condition compared to the waiting list control condition were more likely to dropout at both follow-up points (Table 2). Older participants were less likely to dropout at both follow-up measurements. Men were less likely to dropout at the 6-months follow-up questionnaire. Lastly, participants who were employed compared to unemployed participants were more likely to dropout at both follow-up measurements.

**Table 2:** Results from logistic regression analyses predicting dropout at 6 and 12 months from baseline (*n* = 1127).

Predictors	Dropout at 6 months				Dropout at 12 months			
	OR	SE	95% CI	*p*	OR	SE	95% CI	*p*
Intercept	0.35	0.58	0.11–1.11	0.075	0.80	0.58	0.25–2.52	0.706
Condition^a^	2.72	0.14	2.06–3.60	**<0.001**	2.97	0.14	2.26–3.91	**<0.001**
Gender^b^	0.61	0.14	0.46–0.79	**<0.001**	0.78	0.14	0.60–1.02	0.065
Age	0.99	0.01	0.98–0.998	**0.020**	0.97	0.01	0.96–0.98	**<0.001**
Education low^c^	0.87	0.35	0.44–1.70	0.694	0.77	0.35	0.38–1.51	0.454
Education high^c^	1.03	0.15	0.76–1.40	0.833	1.02	0.15	0.76–1.38	0.887
Marital status^d^	1.13	0.14	0.85–1.49	0.396	1.05	0.14	0.79–1.38	0.757
Work^e^	1.43	0.14	1.08–1.90	**0.012**	1.48	0.14	1.12–1.95	**0.006**
Physical impairment^f^	1.28	0.34	0.65–2.47	0.471	1.36	0.34	0.70–2.65	0.363
BMI	1.02	0.01	0.995–1.05	0.109	1.02	0.01	0.99–1.05	0.154
Health status	1.002	0.001	0.99–1.01	0.743	0.999	0.005	0.99–1.01	0.867
Fruit	1.07	0.06	0.95–1.21	0.264	1.06	0.06	0.94–1.20	0.325
Vegetables	0.998	0.001	0.997–0.999999	0.051	0.999	0.001	0.997–1.001	0.205
Fish	1.02	0.06	0.91–1.15	0.686	1.06	0.06	0.94–1.19	0.370
Unhealthy snacks	0.95	0.04	0.87–1.03	0.201	1.03	0.04	0.95–1.11	0.509
MVPA	1.0001	0.0001	0.9999–1.0002	0.401	1.00003	0.0001	0.9999–1.0002	0.663
R^2^ Tjur	0.080				0.099			

*Note.* Values in bold represent significance after Bonferroni correction (*p* < .025). OR = odds ratio; SE = standard error; 95% CI = 95% Confidence Interval; BMI = body mass index; MVPA = weekly minutes of moderate to vigorous physical activity; R^2^ Tjur = Coefficient of Discrimination.

a The control condition serves as the reference category.

b Women are the reference category.

c Medium education is the reference category.

d Being single is the reference category.

e Unemployed is the reference category.

f Having no physical impairment serves as the reference category.

### 3.3 Main effects on dietary and PA behaviors

The first set of linear mixed-effect models showed that there was a significant time effect for fruit, fish and the consumption frequency of snacks (all *p*-values < .001) but not for vegetables and weekly minutes of MVPA. Results show an increase in fruit and fish intake, and a decrease in snacking frequency at 6- and 12-months post-baseline. However, there was no significant time × condition interaction for any of the dietary and PA behaviors (all *p* values > .02 based on Bonferroni correction), indicating that the intervention group did not perform better than the control condition both at 6- and 12-months post-baseline (Table 3, effects are bold, covariates not). Table S1 in the Supplementary information shows the means and standard deviations for all outcomes for both the research conditions at all time points.

**Table 3:** Linear mixed effect models of the effect of the intervention over time on dietary and PA outcomes.

Predictors	Fruit			Vegetables			Fish			Snacks			MVPA		
	Coef.	95% CI	p	Coef.	95% CI	p	Coef.	95% CI	p	Coef.	95% CI	p	Coef.	95% CI	p
Intercept	1.05	0.97–1.14	<0.001	10.86	10.28–11.45	<0.001	0.61	0.50–0.72	<0.001	1.09	0.99–1.19	<0.001	29.53	27.49–31.56	<0.001
Behavior at 6 months	**0.09**	**0.05–0.14**	**<0.001**	**0.11**	**−0.25–0.47**	**0.541**	**0.18**	**0.12–0.24**	**<0.001**	**−0.12**	**−0.18 to −0.05**	**<0.001**	**1.00**	**−0.17–2.17**	**0.093**
Behavior at 12 months	**0.09**	**0.05–0.14**	**<0.001**	**0.29**	**−0.08–0.65**	**0.122**	**0.14**	**0.08–0.21**	**<0.001**	**−0.11**	**−0.17 to −0.05**	**0.001**	**0.52**	**−0.67–1.72**	**0.393**
Condition^a^	**−0.0005**	**−0.06–0.06**	**0.988**	**−0.24**	**−0.66–0.18**	**0.255**	**0.05**	**−0.03–0.13**	**0.230**	**0.004**	**−0.07–0.07**	**0.905**	**1.23**	**−0.21–2.68**	**0.093**
Gender^b^	−0.08	−0.13 to −0.02	0.004	−0.86	−1.23 to −0.48	<0.001	0.02	−0.05–0.09	0.636	0.08	0.02–0.14	0.012	2.64	1.32–3.96	<0.001
Age	0.004	0.002–0.01	<0.001	0.01	−0.001–0.03	0.068	0.01	0.01–0.01	<0.001	−0.01	−0.01 to −0.003	<0.001	0.07	0.02–0.13	0.006
Marital status^c^	−0.002	−0.06–0.06	0.955	0.10	−0.30–0.50	0.622	0.08	0.01–0.16	0.033	0.03	−0.03–0.10	0.324	−0.58	−1.98–0.82	0.416
Education (low)^d^	−0.004	−0.14–0.13	0.954	0.63	−0.30–1.56	0.185	0.01	−0.17–0.18	0.922	0.13	−0.03–0.28	0.103	1.52	−1.78–4.81	0.367
Education (high)^d^	0.07	0.01–0.13	0.024	1.60	1.18–2.02	<0.001	0.15	0.08–0.23	<0.001	−0.04	−0.11–0.03	0.267	−3.53	−4.99 to −2.06	<0.001
Work^e^	0.01	−0.04–0.07	0.660	−0.05	−0.44–0.34	0.805	−0.05	−0.12–0.02	0.183	−0.10	−0.17 to −0.03	0.003	0.73	−0.65–2.11	0.301
Physical impairment^f^	−0.005	−0.14–0.13	0.945	−0.21	−1.15–0.73	0.663	−0.11	−0.29–0.07	0.217	−0.28	−0.44 to −0.12	<0.001	−6.65	−9.96 to −3.35	<0.001
BMI	−0.01	−0.01–0.0003	0.062	−0.01	−0.04–0.03	0.740	−0.002	−0.01–0.005	0.552	0.002	−0.004–0.01	0.447	−0.22	−0.35 to −0.09	0.001
Health status	0.005	0.001–0.005	0.003	0.03	0.01–0.04	<0.001	0.002	−0.00002–0.005	0.052	−0.003	−0.01 to −0.001	0.002	0.14	0.09–0.18	<0.001
6 months × Condition	**−0.03**	**−0.09–0.02**	**0.249**	**0.14**	**−0.32–0.60**	**0.545**	**−0.07**	**−0.15–0.01**	**0.095**	**−0.01**	**−0.09–0.07**	**0.813**	**0.07**	**−1.44–1.58**	**0.930**
12 months × Condition	**−0.02**	**−0.08–0.03**	**0.413**	**−0.08**	**−0.56–0.39**	**0.727**	**−0.07**	**−0.16–0.01**	**0.086**	**−0.03**	**−0.11–0.06**	**0.530**	**−0.26**	**−1.83–1.30**	**0.741**


Random effects					
σ^2^	0.07	4.71	0.14	0.14	47.51
τ_00_	0.15_id_	6.61_id_	0.25_id_	0.18_id_	84.76_id_
ICC	0.69	0.58	0.63	0.55	0.64
N	1142_id_	1142_id_	1142_id_	1142_id_	1132_id_
Observations	2371	2366	2370	2351	2283
Marginal/Conditional R^2^	0.041/0.704	0.069/0.612	0.075/0.657	0.049/0.575	0.108/0.680

*Note*. Outcomes were square root transformed. Primary outcomes are printed in bold. Coef. = unstandardized coefficient; 95% CI = 95% confidence level; σ^2^ = within-group variance; τ_00_ = between-group-variance; ICC = Intraclass Correlation Coefficient; R^2^ = explained variance; MVPA = moderate-to-vigorous physical activity (minutes per week).

a The wait list control condition is the reference category.

b Women is the reference category.

c Being single is the reference category.

d Medium education is the reference category.

e Being unemployed is the reference category.

f No physical impairment is the reference category.

### 3.4 Interaction with opening session

In the second set of linear mixed effects models, no significant association was found between following the opening session and any of the dietary and PA outcomes in the first step. There was, however, a significant time × opening session interaction effect for fruit intake (6 months: coefficient = 0.14, *p* = .003, 95% CI [0.05–0.24]; 12 months: coefficient = 0.12, *p* = .018, 95% CI [0.02–0.22]). Participants who followed the opening session had a steeper slope (indicating a higher increase in consumption) in fruit intake at 6 months and 12 months compared to baseline than for participants who did not follow the opening session (see Table 4).

**Table 4:** Results from the mixed effect analyses including the opening session.

Predictors	Fruit			Vegetables			Fish			Snacks			MVPA		
	Coef.	95% CI	p	Coef.	95% CI	p	Coef.	95% CI	p	Coef.	95% CI	p	Coef.	95% CI	p
Intercept	1.05	0.97–1.13	<0.001	10.84	10.26–11.43	<0.001	0.61	0.50–0.72	<0.001	1.09	0.99–1.19	<0.001	29.56	27.53–31.59	<0.001
Behavior at 6 months	**0.09**	**0.05–0.14**	**<0.001**	**0.11**	**−0.25–0.47**	**0.540**	**0.18**	**0.12–0.24**	**<0.001**	**−0.12**	**−0.18 to −0.05**	**<0.001**	**1.00**	**−0.16–2.17**	**0.092**
Behavior at 12 months	**0.09**	**0.05–0.14**	**<0.001**	**0.29**	**−0.08–0.65**	**0.121**	**0.14**	**0.08–0.21**	**<0.001**	**−0.11**	**−0.17 to −0.05**	**0.001**	**0.52**	**−0.67–1.72**	**0.391**
Condition^a^	**0.06**	**−0.03–0.14**	**0.221**	**−0.37**	**−1.00–0.26**	**0.252**	**0.13**	**0.01–0.25**	**0.031**	**−0.04**	**−0.15–0.06**	**0.433**	**0.86**	**−1.31–3.03**	**0.439**
Opening session^b^	**−0.07**	**−0.15–0.01**	**0.100**	**0.16**	**−0.43–0.75**	**0.600**	**−0.10**	**−0.21–0.01**	**0.069**	**0.06**	**−0.04–0.16**	**0.249**	**0.47**	**−1.56–2.51**	**0.649**
Gender^c^	−0.08	−0.13 to −0.02	0.006	−0.87	−1.24 to −0.49	<0.001	0.02	−0.05–0.09	0.564	0.08	0.02–0.14	0.014	2.64	1.32–3.96	<0.001
Age	0.004	0.002–0.01	<0.001	0.01	−0.001–0.03	0.065	0.01	0.01–0.01	<0.001	−0.01	−0.01 to −0.003	<0.001	0.07	0.02–0.13	0.006
Marital status^d^	−0.001	−0.06–0.06	0.974	0.13	−0.27–0.53	0.523	0.08	0.005–0.15	0.037	0.04	−0.03–0.10	0.300	−0.61	−2.01–0.79	0.390
Education (low)^e^	−0.001	−0.13–0.13	0.994	0.65	−0.28–1.58	0.168	0.01	−0.16–0.18	0.907	0.13	−0.03–0.28	0.104	1.45	−1.84–4.75	0.387
Education (high)^e^	0.07	0.01–0.13	0.022	1.60	1.19–2.02	<0.001	0.15	0.08–0.23	<0.001	−0.04	−0.11–0.03	0.261	−3.53	−5.00 to −2.07	<0.001
Work^f^	0.01	−0.04–0.07	0.660	−0.05	−0.44–0.35	0.809	−0.05	−0.12–0.02	0.180	−0.10	−0.17 to −0.03	0.003	0.73	−0.66–2.11	0.302
Physical impairment^g^	−0.004	−0.14–0.13	0.955	−0.22	−1.16–0.71	0.639	−0.11	−0.28–0.07	0.228	−0.28	−0.44 to −0.13	<0.001	−6.65	−9.96 to −3.33	<0.001
BMI	−0.01	−0.01–0.0004	0.069	−0.01	−0.04–0.03	0.709	−0.002	−0.01–0.01	0.590	0.002	−0.004–0.01	0.473	−0.22	−0.35 to −0.09	0.001
Health status	0.003	0.001–0.005	0.003	0.03	0.01–0.04	<0.001	0.002	−0.0001–0.005	0.051	−0.003	−0.01 to −0.001	0.002	0.14	0.09–0.18	<0.001
6 months × Condition^a^	−0.15	−0.25 to −0.06	0.002	−0.61	−1.40–0.19	0.135	−0.13	−0.27–0.01	0.076	0.02	−0.12–0.16	0.770	2.26	−0.37–4.89	0.093
12 months × Condition^a^	−0.13	−0.23 to −0.02	0.017	−0.85	−1.70 to −0.01	0.046	−0.15	−0.30 to −0.00	0.047	−0.02	−0.17–0.13	0.807	1.34	−1.45–4.13	0.347
6 months × Opening session^b^	**0.14**	**0.05–0.24**	**0.003**	**0.90**	**0.12–1.67**	**0.024**	**0.07**	**−0.07–0.21**	**0.307**	**−0.04**	**−0.17–0.10**	**0.592**	**−2.63**	**−5.21 to −0.05**	**0.046**
12 months × Opening session^b^	**0.12**	**0.02–0.22**	**0.018**	**0.92**	**0.09–1.75**	**0.029**	**0.09**	**-0.05–0.24**	**0.208**	**−0.01**	**−0.16–0.13**	**0.879**	**−1.94**	**−4.69–0.81**	**0.167**


Random effects					
σ^2^	0.07	4.67	0.14	0.14	47.27
τ_00_	0.15_id_	6.62_id_	0.24_id_	0.18_id_	85.02_id_
ICC	0.69	0.59	0.63	0.55	0.64
N	1142_id_	1142_id_	1142_id_	1142_id_	1132_id_
Observations	2371	2366	2370	2351	2283
Marginal R^2^/Conditional R^2^	0.043/0.706	0.073/0.616	0.076/0.658	0.049/0.575	0.109/0.682

*Note*. Outcomes were square root transformed. Primary outcomes are printed in bold. Coef. = unstandardized coefficient; 95% CI = 95% confidence level; σ^2^ = within-group variance; τ_00_ = between-group-variance; ICC = Intraclass Correlation Coefficient; R^2^ = explained variance; MVPA = moderate-to-vigorous physical activity (minutes per week).

a The waiting list control condition is the reference category.

b No opening session is the reference category.

c Women is the reference category.

d Being single is the reference category.

e Medium education is the reference category.

f Being unemployed is the reference category.

g No physical impairment is the reference category.

### 3.5 Interaction with session usage

In the third set of analyses, we found that the number of completed sessions in the diet and PA module was not significantly associated with any of the outcomes (all *p* values > .01). However, there were significant time × diet module session interactions for fruit intake, vegetable intake, and the daily consumption frequency of unhealthy snacks (fruit: coefficient = 0.03, *p* = .002, 95% CI [0.01–0.05]; vegetables: coefficient = 0.22, *p* = .003, 95% CI [0.08–0.37]; snacks: −0.03, *p* = .008, 95% CI [−0.06 to −0.01]). The results can be seen in Table 5. More completed dietary sessions were associated with steeper positive slopes (i.e., higher increase) over time for fruit and vegetables 6 months post-baseline. Furthermore, more completed dietary sessions were associated with a steeper negative slope over time in the consumption frequency of unhealthy snacks. Thus, participants who followed more sessions in the diet module had a higher fruit and vegetables intake at the 6 months follow-up measurement and a lower daily snacking frequency at the 12 months follow-up measurement. Tables S2–4 in the Supplementary information display the raw means for fruit and vegetable intake, and the consumption frequency of unhealthy snacks stratified for the diet sessions, including the slope. This information further illustrates the effects (slopes) of sessions over time for the three outcomes.

**Table 5:** Results from the mixed effect analyses including number of sessions within diet and PA module.

Predictors	Fruit			Vegetables			Fish			Snacks			MVPA		
	Coef.	95% CI	p	Coef.	95% CI	p	Coef.	95% CI	p	Coef.	95% CI	p	Coef.	95% CI	p
Intercept	1.07	0.99–1.15	<0.001	10.92	10.34–11.49	<0.001	0.63	0.53–0.74	<0.001	1.08	0.99–1.18	<0.001	29.68	27.68–31.68	<0.001
Behavior at 6 months	**0.05**	**0.02–0.08**	**0.002**	**−0.01**	**−0.27–0.25**	**0.926**	**0.13**	**0.08–0.17**	**<0.001**	**−0.10**	**−0.15 to −0.06**	**<0.001**	**0.92**	**0.11–1.73**	**0.025**
Behavior at 12 months	**0.06**	**0.03–0.10**	**<0.001**	**0.07**	**−0.20–0.34**	**0.610**	**0.09**	**0.04–0.14**	**<0.001**	**−0.09**	**−0.14 to −0.05**	**<0.001**	**0.25**	**−0.60–1.10**	**0.562**
Sessions Diet	**−0.02**	**−0.04–0.01**	**0.215**	**0.05**	**−0.14–0.24**	**0.594**	**0.01**	**−0.03–0.04**	**0.667**	**0.01**	**−0.02–0.04**	**0.672**	**0.19**	**−0.42–0.81**	**0.535**
Sessions PA	**0.003**	**−0.03–0.03**	**0.842**	**−0.02**	**−0.22–0.18**	**0.847**	**0.01**	**−0.03–0.05**	**0.545**	**0.01**	**−0.02–0.05**	**0.390**	**−0.66**	**−1.41–0.09**	**0.083**
Condition^a^	**0.004**	**−0.08–0.09**	**0.934**	**−0.64**	**−1.22 to −0.06**	**0.030**	**0.07**	**−0.03–0.18**	**0.183**	**−0.04**	**−0.14–0.06**	**0.436**	**1.38**	**−0.65–3.40**	**0.182**
Opening session^b^	**−0.02**	**−0.11–0.06**	**0.612**	**0.28**	**−0.30–0.86**	**0.342**	**−0.10**	**−0.21–0.01**	**0.065**	**0.05**	**−0.05–0.15**	**0.337**	**−0.10**	**−2.13–1.94**	**0.926**
Gender^c^	−0.08	−0.13 to −0.02	0.005	−0.85	−1.23 to −0.48	<0.001	0.02	−0.05–0.09	0.511	0.08	0.02–0.14	0.014	2.64	1.32–3.96	<0.001
Age	0.004	0.002–0.01	<0.001	0.01	−0.001–0.03	0.066	0.01	0.01–0.01	<0.001	−0.01	−0.01 to −0.003	<0.001	0.08	0.02–0.13	0.005
Marital status^d^	−0.003	−0.06–0.05	0.915	0.15	−0.25–0.55	0.466	0.08	0.01–0.16	0.030	0.04	−0.03–0.10	0.299	−0.62	−2.03–0.78	0.383
Education low^e^	−0.005	−0.14–0.13	0.943	0.61	−0.31–1.54	0.195	0.01	−0.17–0.18	0.921	0.13	−0.02–0.29	0.091	1.40	−1.89–4.70	0.404
Education high^e^	0.07	0.01–0.13	0.024	1.60	1.18–2.01	<0.001	0.15	0.08–0.23	<0.001	−0.04	−0.11–0.03	0.267	−3.51	−4.97 to −2.05	<0.001
Work^f^	0.01	−0.04–0.07	0.650	−0.04	−0.44–0.35	0.829	−0.05	−0.12–0.03	0.205	−0.10	−0.16 to −0.03	0.003	0.66	−0.73–2.04	0.353
Impairment^g^	−0.003	−0.14–0.13	0.966	−0.29	−1.23–0.65	0.550	−0.12	−0.30–0.06	0.180	−0.29	−0.44 to −0.13	<0.001	−6.46	−9.78 to −3.14	<0.001
BMI	−0.01	−0.01–0.0003	0.063	−0.01	−0.05–0.03	0.669	−0.002	−0.01–0.005	0.546	0.002	−0.004–0.01	0.479	−0.22	−0.35 to −0.08	0.001
Health status	0.003	0.001–0.005	0.003	0.03	0.01–0.04	<0.001	0.002	−0.0001–0.005	0.061	−0.004	−0.01 to −0.001	0.001	0.14	0.10–0.19	<0.001
Time (6 months) × Sessions diet	**0.03**	**0.01–0.05**	**0.002**	**0.22**	**0.08–0.37**	**0.003**	**0.01**	**−0.01–0.04**	**0.315**	**−0.02**	**−0.05–0.001**	**0.067**			
Time (12 months) × Sessions diet	**0.02**	**0.002–0.04**	**0.031**	**0.17**	**0.03–0.32**	**0.021**	**0.01**	**−0.02–0.03**	**0.556**	**−0.03**	**−0.06 to −0.01**	**0.008**			
Time (6 months) × Sessions PA													**0.25**	**−0.30–0.80**	**0.370**
Time (12 months) × Sessions PA													**0.25**	**−0.31–0.80**	**0.379**


Random effects					
σ^2^	0.07	4.68	0.14	0.14	47.50
τ_00_	0.15_id_	6.56_id_	0.24_id_	0.18_id_	84.48_id_
ICC	0.69	0.58	0.63	0.55	0.64
N	1142_id_	1142_id_	1142_id_	1142 _id_	1132_id_
Observations	2371	2366	2370	2351	2283
Marginal R^2^/Conditional R^2^	0.0421/0.705	0.076/0.615	0.078/0.656	0.051/0.577	0.110/0.680

*Note*. Outcomes were square root transformed. Primary outcomes are printed in bold. Coef. = unstandardized coefficient; 95% CI = 95% confidence level; σ^2^ = within-group variance; τ_00_ = between-group-variance; ICC = Intraclass Correlation Coefficient; R^2^ = explained variance; MVPA = moderate-to-vigorous physical activity (minutes per week).

a The waiting list control condition is the reference category.

b No opening session is the reference category.

c Women is the reference category.

d Being single is the reference category.

e Medium education is the reference category.

f Being unemployed is the reference category.

g No physical impairment is the reference category.

## 4 Discussion

The current study examined the effects of MyLifestyleCoach, an intervention based on SDT/MI to promote dietary and PA behavior in Dutch adults, compared to a waiting list control condition. The results of the primary analysis in our RCT showed that the intervention was not effective in improving components of dietary intake and PA. This is not in line with the findings of the study by Friederichs et al., 2015, Friederichs et al., 2016 with regard to PA and the generally proven effectiveness of other CT interventions on the two behaviors as concluded in several reviews (Broekhuizen et al., 2012; Kroeze et al., 2006; Lustria et al., 2013; Neville et al., 2009). The latter reviews demonstrate that the successful interventions were predominantly guided by Transtheoretical Model and Social Cognitive Theory. It is worth mentioning that interventions using SDT/MI were relatively scarce at that moment and they were not included in these reviews for that reason. It also has to be noted that the studies included in these reviews differ on other aspects from our study, such as delivery mode and number of behaviors addressed (many single behavior studies). This makes it difficult to compare our findings to these studies. Further in-depth analyses showed that there was a stronger increase in fruit consumption over time in the group of participants who followed the opening session compared to the group of participants who did not follow the opening session. In addition, participants who completed more sessions in the diet module display a stronger increase in fruit and vegetable consumption, and a stronger decrease in the consumption frequency of unhealthy snacks over time.

One of the explanations that there was no overall effect of the intervention on the dietary behaviors could be that we limited our study to four primary dietary outcomes where participants might have formulated other dietary goals in their action plan (e.g., other behaviors or in terms of frequency or amount). Dietary behavior is complex. It consists of many (sub-)behaviors, such as the consumption of fruit and vegetables (Coumans et al., 2021a). Participants who followed the diet module could make an action plan in which they formulated their personal dietary goals. It could be that these goals were different from the dietary behaviors we measured as outcomes of this study, and for that reason, our outcome measures might have been too general to detect improvements regarding personal goals. A future study could examine to what extent the findings differ when the personal goals of participants are taken into account.

Another explanation for the absence of effects on dietary and PA behaviors could be that many participants made no or limited use of the intervention. Possibly, they were not interested in the module(s) which resulted in selecting the option in the opening session that they did not want to follow any module in the opening session. Further, some have dropped out before the intervention started. Thus, they did not engage in the opening session and subsequently could not continue to the rest of the intervention, or they dropped-out during the intervention period. Reasons for this could be that the intervention features did not match participant's prerequisites or that the participants were already satisfied with the first parts of the intervention, such as feedback on their behavior or creating an action plan, and did not expect to further benefit from the intervention (Johansson et al., 2015; Vandereycken and Devidt, 2010).

As mentioned earlier, when intervention usage was considered, we found a stronger increase in fruit consumption at 6- and 12-months post-baseline for the group of participants who followed at least the intervention's opening session compared with the group of participants who did not follow the opening session. The opening session had the purpose of engaging participants with the intervention and its topics, while the participants were free to make their own choices. Feedback about their current dietary and PA behavior was also provided in a non-judgmental way, in line with the SDT/MI approach. For some participants this short session with awareness-raising feedback might already have been sufficient to change their fruit intake in this study (Teasdale et al., 2018; van Stralen et al., 2011). However, these findings should be interpreted with caution. The opening session was obligatory to take part in the intervention and the effects found here could also be attributable to those participants who used subsequent modules.

In addition, the analyses showed that people who followed more sessions within the diet module increased their fruit and vegetable intake more strongly in the short-term. This is in line with the hypotheses. These findings regarding behavior change are in line with a review and a meta-analysis. This research showed the efficacy of MI, generally in face-to-face settings or by telephone and only in a few cases by email but not online, as its use was associated with increased fruit and vegetable consumption, with MI also having sustained effects (Burke et al., 2003; Martins and McNeil, 2009). This SDT/MI approach seems particularly promising for the opening session on the short- and long-term, and the intensity of diet module's sessions seems promising on the short-term.

Furthermore, the results demonstrated that people who followed more sessions within the diet module had a steeper decline in unhealthy snacks in the long term, but not in the shorter period. The opening session was insufficient to induce behavior change for this outcome, but the (more intensive use of the) elaborative SDT/MI sessions seem(s) essential to decrease snacking behavior. This explanation should be interpreted with caution as participants were not randomized to conditions with different intervention doses, but chose themselves to use the intervention more intensively or to follow certain modules. These participants might be more motivated to change their behavior and use interventions more intensively (e.g., Chinn et al., 2006). Interventions that specifically target unhealthy snacks are relatively scarce. Still, as snacks are generally assumed to be highly caloric or high in sugars and fat, our finding is in line with Broekhuizen and colleagues' review. They showed an improvement in fat consumption in 17 out of 21 studies in favor of the CT interventions with small effect sizes for short, medium and long-term follow-up.

Snacking is often seen as a hardwired unhealthy habit. Many people have the intention to consume fewer unhealthy snacks, but they often fail to do so (Kumanyika et al., 2000). More sessions (of MI) might be necessary to help participants to change their behavior. As snacking behavior can be considered a habit or an automatic behavior performed unintentionally and with little controllability, making participants actively deal with their “unwanted” behavior through multiple MI-based sessions could aid behavior change (Aarts et al., 1998). Thus, unlearning a certain habit might take more time to change than learning a new behavior. Furthermore, it could be that the effects of an increase in fruit and vegetable intake in our study are linked to a decrease in snacking behavior, because they eat more fruit and vegetables, they eat fewer snacks. Due to the nature of this study, we can only conclude that following more sessions is beneficial for behavior change. We cannot provide detailed information about what specific elements of the intervention are crucial to achieve behavior change. For future studies, it would be relevant to assess which factors would contribute to behavior change.

Although the intervention was also developed to increase PA behavior, the finding that people did not improve their weekly minutes of MVPA is not surprising. A plausible explanation can be found in our target group of participants. Most of the intervention's participants (94.9%) adhered to the guideline of 150 min of MVPA per week at the baseline assessment. The previous version of the PA module (*I Move*), which was effective in increasing weekly minutes of MVPA, explicitly focused on participants who still were limited in their weekly minutes of MVPA (Friederichs et al., 2015, Friederichs et al., 2016). Our intervention focused more broadly on participants who were interested in a healthy lifestyle with no exclusion criteria on diet and PA, and consequently, our target population was different (Friederichs et al., 2014). One consequence of our targeted population could be that they were already more engaged in sufficient PA. Thus, there was little room for improvement in their PA behavior.

From the literature we know that more autonomous forms of motivation are generally linked to behavior change (Teixeira et al., 2012; Hagger and Chatzisarantis, 2008; Verstuyf et al., 2012; Pelletier and Dion, 2007; Fortier et al., 2007; McSpadden et al., 2016; Shaikh et al., 2008; Trudeau et al., 1998). One of the intervention's aims was to create a context to foster the basic psychological needs to promote more autonomous forms of motivation. To develop a full picture of the underlying mechanisms of behavior change in this intervention, additional studies are necessary to investigate whether motivation served as such a mechanism. Moderation analyses could further identify for which specific participant subgroups, such as gender-related, the intervention exerted its greatest effects.

### 4.1 Strengths and limitations

This study is one of the first to examine the short- and long-term effects of a diet and PA web-based intervention based on SDT and MI, in which participants were free to choose on what behavior to work on. But there are several limitations of this study that should be noted. First, although validated instruments (or instruments based on validated instruments, such as the FFQ) were used to assess dietary and PA behavior, they were still self-reported and therefore vulnerable to biases, such as social desirability (e.g., over reporting of PA or diet intake). Future interventions that focus on diet and PA behavior could be complemented with more objective measures (Silfee et al., 2018; Naska et al., 2017). In addition, individuals could have the possibility to fill in the baseline questionnaire multiple times and thereby taking part in the intervention twice or more. However, this risk was small due to preventive measures such as being to register with only one e-mail address. This issue will not likely have influenced the study's results.

Furthermore, one of the major challenges in the field of eHealth is the high number of people who do not complete the intervention as intended (“non-usage attrition”) or do not complete follow-up questionnaires (“dropout attrition”) (Eysenbach, 2005). As autonomy support is central to SDT/MI, providing participants with choices, for example, which behavior to work on and when to start with the chosen module(s), could lead to better engagement and usage (Markland et al., 2005). Despite implementing autonomy in several ways, such as offering choice in the opening session, both non-usage and dropout attrition were still considerable. Possibly, it required more time and effort to participate in the intervention than expected. Further research should be undertaken to investigate why certain participants did not follow the opening session or why they stopped using the intervention.

Nonetheless, people randomized into the intervention condition were less likely to complete the follow-up questionnaires compared to people randomized into the waiting list control condition. This can be interpreted as a threat to the internal validity of this study—i.e., did the intervention really induce behavioral change? It could be that people in the intervention condition who did not complete the follow-up questionnaire(s) did not change their behavior and intervention effects could be overestimated as we did not apply an intention to treat procedure in our analysis.

In line with this limitation, the results of this study could have been influenced by selective dropout. For instance, younger participants were less likely to complete the follow-up questionnaires than older participants. This was taken into account by including the predictors of selective dropout as control variables in the main analyses and by applying mixed effect models, to deal with the missing data in the most accurate way possible (Twisk, 2013). Still, the findings should be interpreted with caution, as it concerns a self-selected group of participants.

Last, the participants were recruited using a research panel. One of the advantages of using a research panel, is that you can attract and recruit specific groups. Initially, we aimed to recruit especially people with a low educational level. Despite this effort, still a relatively large group of people with a higher educational level and participants who already engaged in sufficient PA were over represented in this work, which unfortunately is common in these types of studies (Rhodes et al., 2003; Cheung et al., 2017). More efforts need to be made to obtain a representative study population with sufficient low educated participants and those who do not meet the guidelines for a healthy diet or PA.

### 4.2 Implications

With the standard RCT approach that has been used, we found no overall intervention effects. However, the intervention shows promise for those who chose to use the intervention more intensively. It therefore seems to be important to improve intervention use. This would require finding a better balance between providing freedom of choice and autonomy in which intervention elements to use (in line with SDT) and making sure that people are exposed to necessary intervention content. More studies are needed, and several adaptations must be realized before the intervention is ready for implementation into practice. Suggested improvements include optimizing content and preventing drop-out on specific moments in the intervention. For example, we advise to offer content that is more relevant to the participants, by using even more personalization (for example in language and use of multimedia) to achieve a better intervention fit. A second example is to use more genuine SDT/MI strategies such as expressing empathy or promoting self-efficacy from the moment we approach the target group to participate in the intervention (Coumans et al., 2021b; Shingleton and Palfai, 2016; Galvao Gomes da Silva et al., 2020). In addition, it is useful to examine the effectiveness of the added value of SDT/MI or combining diet and PA behavior in an intervention versus focusing on a single behavior in another study.

## 5 Conclusion

This web-based CT intervention based on SDT/MI to promote dietary behavior was not effective in inducing behavior change in case we applied a conventional RCT analysis. In the in-depth analyses in which self-chosen intervention use was taken into account, there were indications for a beneficial effect for participants who attended the opening session and more intervention sessions. Future studies are necessary to further identify for which specific participant subgroups the intervention exerted its greatest effects and what strategies could be implemented to increase overall intervention usage and effects.

## Ethics approval and consent to participate

This RCT has been ethically approved by the Committee for Ethics and Consent in Research of the Open University of the Netherlands (reference number: U2018/07266/SVW). All participants signed an online informed consent form.

## Consent for publication

Not applicable.

## Availability of data and materials

The datasets generated during and/or analyzed during the current study are available from the corresponding author on reasonable request.

## Funding sources

The study was funded by an internal fund of the Open University of the Netherlands.

## Statement of authorship

JC wrote the manuscript and performed the data analysis. All authors had the idea for the study and take full responsibility for and have read and approved this final version of this manuscript.

## Declaration of competing interest

The authors declare that they have no known competing financial interests or personal relationships that could have appeared to influence the work reported in this paper.

## Appendix A Supplementary data

Supplementary tables Image 1
