# Supplementary material for: The effects of a web-based computer-tailored diet and physical activity intervention based on self-determination theory and motivational interviewing: A randomized controlled trial
Source: Internet Interv. 2022 Apr 14;28:100537. doi: 10.1016/j.invent.2022.100537 (PMC9058957; doi:10.1016/j.invent.2022.100537)
Supplement: Supplementary file 1 — Supplementary tables [file mmc1.docx]

**Supplementary information**

**Table S1** Means and standard deviations for the outcomes at baseline, 6 months, and 12 months

| Variables | Group | Baseline  (mean (SD)) | n | 6 months  (mean (SD)) | n | 12 months  (mean (SD)) | n |
| --- | --- | --- | --- | --- | --- | --- | --- |
| Fruit intake (portions/day) | Intervention | 1.4 (1.1) | 775 | 1.5 (1.1) | 381 | 1.5 (1.0) | 335 |
|  | Control | 1.4 (1.0) | 367 | 1.6 (1.2) | 262 | 1.6 (1.1) | 251 |
| Vegetable intake (grams/day) | Intervention | 143.1 (80.7) | 775 | 151.6 (84.5) | 380 | 152.1 (87.5) | 335 |
|  | Control | 148.4 (83.9) | 367 | 151.2 (80.9) | 260 | 158.0 (85.9) | 249 |
| Fish (portions/week) | Intervention | 1.1 (1.1) | 775 | 1.2 (1.1) | 381 | 1.2 (1.1) | 335 |
|  | Control | 1.0 (1.0) | 367 | 1.3 (1.2) | 261 | 1.2 (1.2) | 251 |
| Unhealthy snacks (consumption frequency per day) | Intervention | 1.5 (1.9) | 775 | 1.2 (1.7) | 376 | 1.1 (1.1) | 327 |
|  | Control | 1.4 (1.4) | 367 | 1.3 (1.6) | 260 | 1.2 (1.4) | 246 |
| MVPA (minutes/week) | Intervention | 992.7 (836.8) | 767 | 1034.1 (792.2) | 357 | 997.4 (800.5) | 313 |
|  | Control | 941.6 (848.7) | 360 | 999.4 (870.6) | 250 | 962.4 (782.9) | 236 |

**Table S2** Absolute values for the fruit intake (portions per day) stratified over the number of sessions in the diet module

| Number of sessions diet module | Baseline | 6 months | Difference 6 months and baseline | n _0months_ | n _6months_ |
| --- | --- | --- | --- | --- | --- |
| 0 | 1.4 | 1.5 | 0.12 | 856 | 475 |
| 1 | 1.3 | 1.1 | -0.06 | 82 | 17 |
| 2 | 1.3 | 1.3 | 0.24 | 36 | 9 |
| 3 | 1.4 | 1.7 | 0.23 | 41 | 23 |
| 4 | 1.3 | 1.7 | 0.38 | 127 | 119 |

*Note*. The differences (slopes) were calculated with the missing values removed.

**Table S3** Absolute values for the vegetable intake (grams per day) stratified over the number of sessions in the diet module

| Number of sessions diet module | Baseline | 6 months | Difference 6 months and baseline | n _0months_ | n _6months_ |
| --- | --- | --- | --- | --- | --- |
| 0 | 146 | 147 | -0.1 | 856 | 472 |
| 1 | 140 | 147 | -16 | 82 | 17 |
| 2 | 127 | 137 | 47 | 36 | 9 |
| 3 | 157 | 189 | 14 | 41 | 23 |
| 4 | 143 | 164 | 20 | 127 | 119 |

*Note*. The differences (slopes) were calculated with the missing values removed.

**Table S4**

Absolute values for the consumption frequency of unhealthy snacks per day stratified over the number of sessions in the diet module

| Number of sessions in diet module | Baseline | 12 months after baseline | Difference (slope) | n _0months_ | n _12months_ |
| --- | --- | --- | --- | --- | --- |
|  |  |  |  |  |  |
| 0 | 1.44 | 1.16 | -0.20 | 856 | 411 |
| 1 | 1.21 | 0.87 | -0.01 | 82 | 15 |
| 2 | 1.32 | 0.95 | -0.14 | 36 | 8 |
| 3 | 1.53 | 0.98 | -0.29 | 41 | 25 |
| 4 | 1.64 | 1.01 | -0.63 | 127 | 114 |

*Note*. The differences (slopes) were calculated with the missing values removed.
